# Cross-seeding of alpha-synuclein aggregation by amyloid fibrils of food proteins

**DOI:** 10.1016/j.jbc.2021.100358

**Published:** 2021-02-02

**Authors:** Jonathan Vaneyck, Ine Segers-Nolten, Kerensa Broersen, Mireille M.A. E. Claessens

**Affiliations:** 1 Nanobiophysics, MESA+ Institute for Nanotechnology, University of Twente, Enschede, the Netherlands; 2 Applied Stem Cell Technologies, Technical Medical Centre, University of Twente, Enschede, the Netherlands

**Keywords:** α-synuclein, atomic force microscopy, food proteins, seeding mechanism, heterologous seeding, Parkinson's disease, amyloid fibril, Aβ, amyloid-β, AF, Alexa Fluor, AFM, atomic force microscopy, aSyn, α-synuclein, B100, polymorph at 100 mM NaCl, B13, beta-lactoglobulin polymorph at 13 mM NaCl, L, lysozyme, NAC, Lewy bodies nonabeta component, OD, optical density, PD, Parkinson's disease, ThT, thioflavin-T

## Abstract

The aggregation of the protein α-synuclein (aSyn) into amyloid fibrils in the human brain is associated with the development of several neurodegenerative diseases, including Parkinson's disease. The previously observed prion-like spreading of aSyn aggregation throughout the brain and the finding that heterologous cross-seeding of amyloid aggregation occurs *in vitro* for some proteins suggest that exposure to amyloids in general may pose a risk for disease development. To elucidate which protein fibril characteristics determine if and how heterologous amyloid seeding can occur, we investigated the potential of amyloid fibrils formed from proteins found in food, hen egg white lysozyme, and bovine milk β-lactoglobulin to cross-seed aSyn aggregation in the test tube. We observed that amyloid fibrils from lysozyme, but not β-lactoglobulin, potently cross-seeded the aggregation of aSyn as indicated by a significantly shorter lag phase of aSyn aggregation in the presence of lysozyme fibrils. The cross-seeding effect of lysozyme was found to be primarily driven by a surface-mediated nucleation mechanism. The differential seeding effect of lysozyme and β-lactoglobulin on aSyn aggregation could be explained on the basis of binding affinity, binding site, and electrostatic interactions. Our results indicate that heterologous seeding of proteins may occur depending on the physicochemical characteristics of the seed protein fibril. Our findings suggest that heterologous seeding has the potential to determine the pathogenesis of neurodegenerative amyloid diseases.

Parkinson's disease (PD) is a neurodegenerative protein aggregation disease (1). In this disease, the soluble protein α-synuclein (aSyn) self-assembles into characteristic insoluble β-sheet rich amyloid fibrils that accumulate in so-called Lewy bodies inside neuronal cells of the brain (2). In diseased postmortem brain tissue, Lewy body pathology is accompanied by a loss of dopaminergic neurons. Although PD-related aSyn amyloid fibril formation has long been presumed to occur independently in affected cells, studies indicate that cell-to-cell transmission may play an important role in spreading of the disease (3, 4). These observations culminated in the idea that as the aggregation of aSyn into amyloid fibrils is a nucleation-dependent process, transmitted fibrils may act as nuclei and initiate aSyn aggregation in the recipient cell. Recently, studies showed that aSyn can indeed exert prion-like behavior which may play a role in the development of PD and other synucleopathies (5, 6, 7). The transmission mechanism of aSyn aggregation may not be limited to exposure of recipient cells to aSyn amyloid fibrils or fibril seeds. Besides induction of amyloid fibril formation by proteins with the same sequence, or homologous seeding, amyloid fibrils from other proteins have been shown to induce aggregation of heterologous protein sequences, which has been termed cross-seeding (8, 9, 10, 11).

The first step of the aggregation of proteins into amyloid fibrils, the nucleation or lag phase, represents the assembly of a monomeric protein into an aggregation prone nucleus from which the fibril can grow (12). This nucleation phase can be surpassed by the addition of preformed fibrils also called seeds (12, 13). Even though homologous seeding is generally considered to be more efficient, cases of heterologous seeding have been reported between amyloid-β (Aβ) peptide and tau (14), Aβ and aSyn (15), and Aβ and prion protein (16), as well as between different mutant disease-related aSyn seeds and wild-type aSyn (17), or aSyn seeds and prion protein (18). It is currently not understood why heterologous seeding occurs in some cases and not in others, and it is unknown whether nondisease–related protein seeds can induce heterologous seeding of pathogenic proteins.

During their life span, humans are exposed to amyloid fibrils and fragments thereof from different sources. Nondisease–related amyloid fibrils have been used as building blocks for new materials (19), are biologically active in various life forms including bacteria (20, 21), and are naturally present in foods or used as functional food ingredients (22, 23, 24, 25, 26, 27). It is of interest to investigate the potential of such nondisease–related proteins to cross-seed amyloid formation of a disease-related protein to obtain a better understanding of the biochemical or physicochemical requirements for the process of heterologous seeding but also in view of potential health risks.

We here address if disease-related aSyn can be seeded by the addition of preformed fibril seeds from the nondisease–related proteins lysozyme and β-lactoglobulin in the test tube in buffer solution. The results show that lysozyme seeds, but not β-lactoglobulin seeds, potently induce the aggregation of aSyn into amyloid fibrils. The observed heterologous seeding activity of lysozyme is driven by a fibril surface-mediated nucleation process. The 140 amino acid sequence of aSyn can be divided into three regions: an amphipathic N-terminal region (amino acids 1–60), a hydrophobic central region (called nonabeta component or NAC, amino acids 61–95), and a negatively charged C-terminal region (amino acids 96–140) (28, 29). Our results show that selective seeding by lysozyme but not β-lactoglobulin fibrils seems to be driven by the net positive charge of the lysozyme fibrils causing aSyn to bind *via* its negatively charged C-terminal region leaving the aggregation prone NAC domain solvent exposed. ASyn can also bind the net negatively charged β-lactoglobulin fibril but in this interaction the NAC domain is involved and thus shielded from the solution. This prevents nucleation of aSyn aggregation on the surface of β-lactoglobulin fibrils.

Our results highlight that the aggregation of disease-related amyloidogenic proteins can be heterologously seeded by nondisease–related proteins that share no homology and that the cross-seeding ability depends on the physicochemical properties of the seed.

## Results

In this study, the heterologous seeding of aSyn aggregation by amyloid fibrils composed of the amyloidogenic food proteins hen egg white lysozyme and bovine milk β-lactoglobulin is investigated.

### Aggregation of food proteins into amyloid fibrils

**Figure 1:**
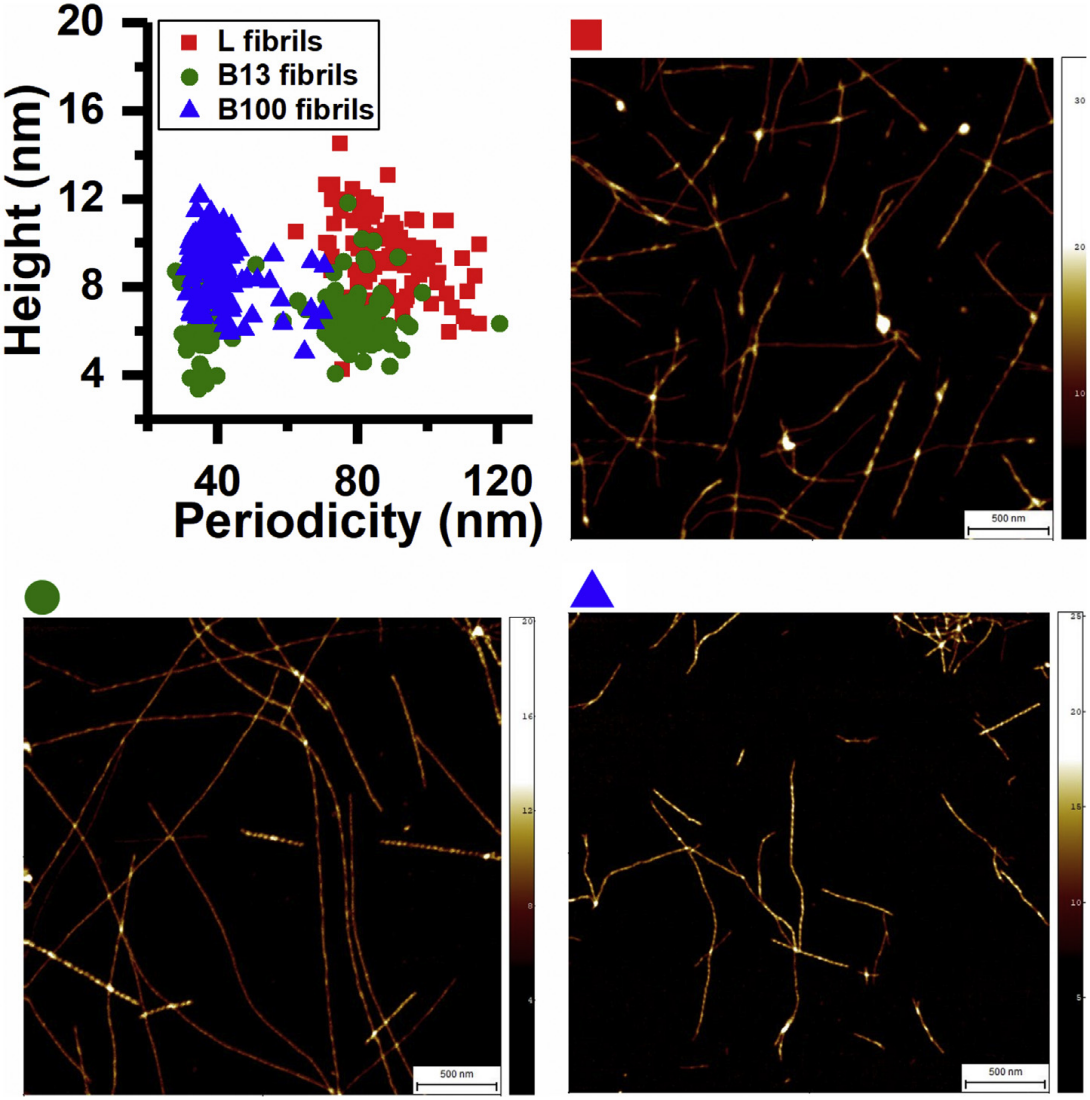
**Amyloid fibrils are formed upon aggregation of lysozyme and β-lactoglobulin monomers**. Height *versus* periodicity plot and AFM images of lysozyme fibrils (*red squares*), β-lactoglobulin polymorph B13 (*green circles*), and polymorph B100 (*blue triangles*) fibrils. The scale bar is 500 nm, and the height scale varies from 0 to 20 to 35 nm.

We started by aggregating hen egg white lysozyme and bovine milk β-lactoglobulin to produce amyloid fibrils that served as a source of seeds upon sonication. Previously, the seeding capacity of amyloid fibrils was reported to depend on structural compatibility of seeds and monomers (17). Therefore, we included two morphologically distinct β-lactoglobulin amyloid fibril populations (polymorphs) by aggregating the protein in a 13 mM NaCl (polymorph “B13”) and in a 100 mM NaCl (polymorph “B100”) containing buffer, as previously reported (30). The resulting lysozyme and β-lactoglobulin aggregates bind the amyloid specific dye thioflavin-T (ThT) (Fig. S1), indicating that the food proteins organized into amyloid structures. Atomic force microscopy (AFM) images (Fig. 1) confirm that these amyloid structures are fibrils. Analysis of AFM images shows that under the aggregation conditions used, lysozyme fibrils vary in height between 4 and 12 nm with a periodicity of 80 to 120 nm. β-Lactoglobulin polymorph B13 fibrils show heights between 4 and 8 nm distributed over two populations with different periodicities: (i) with an average periodicity around 40 nm and (ii) with an average periodicity around 80 nm. Approximately 90% of the observed β-lactoglobulin polymorph B100 fibrils vary in height between 6 and 12 nm with an average periodicity of around 40 nm.

### Fibril seeds of lysozyme, but not β-lactoglobulin, can cross-seed aggregation of aSyn

The ability of amyloid fibrils to heterologously seed aSyn aggregation is reflected in the aSyn aggregation kinetics. Seeding is visible as a reduction of the lag phase duration compared to unseeded aggregation (31).

To address the question if fibrils composed of food proteins can heterologously seed the aggregation of aSyn, we incubated WT aSyn with small quantities of β-lactoglobulin or lysozyme fibril seeds that were generated by exposure of β-lactoglobulin and lysozyme fibrils to a sonication procedure. Judging from the increase in ThT fluorescence with time (Fig. S2*A*), the aggregation of WT aSyn (lag phase 41 ± 10 h) could not be cross-seeded by either of the β-lactoglobulin seed polymorphs (lag phase 47.6 ± 17 h for B100; data for B13 not shown), whereas the presence of lysozyme seeds significantly reduced the duration of the lag phase of WT aSyn aggregation (lag phase 19.5 ± 1.7 h).

### Heterologous seeding is based on a surface-mediated mechanism

**Figure 2:**
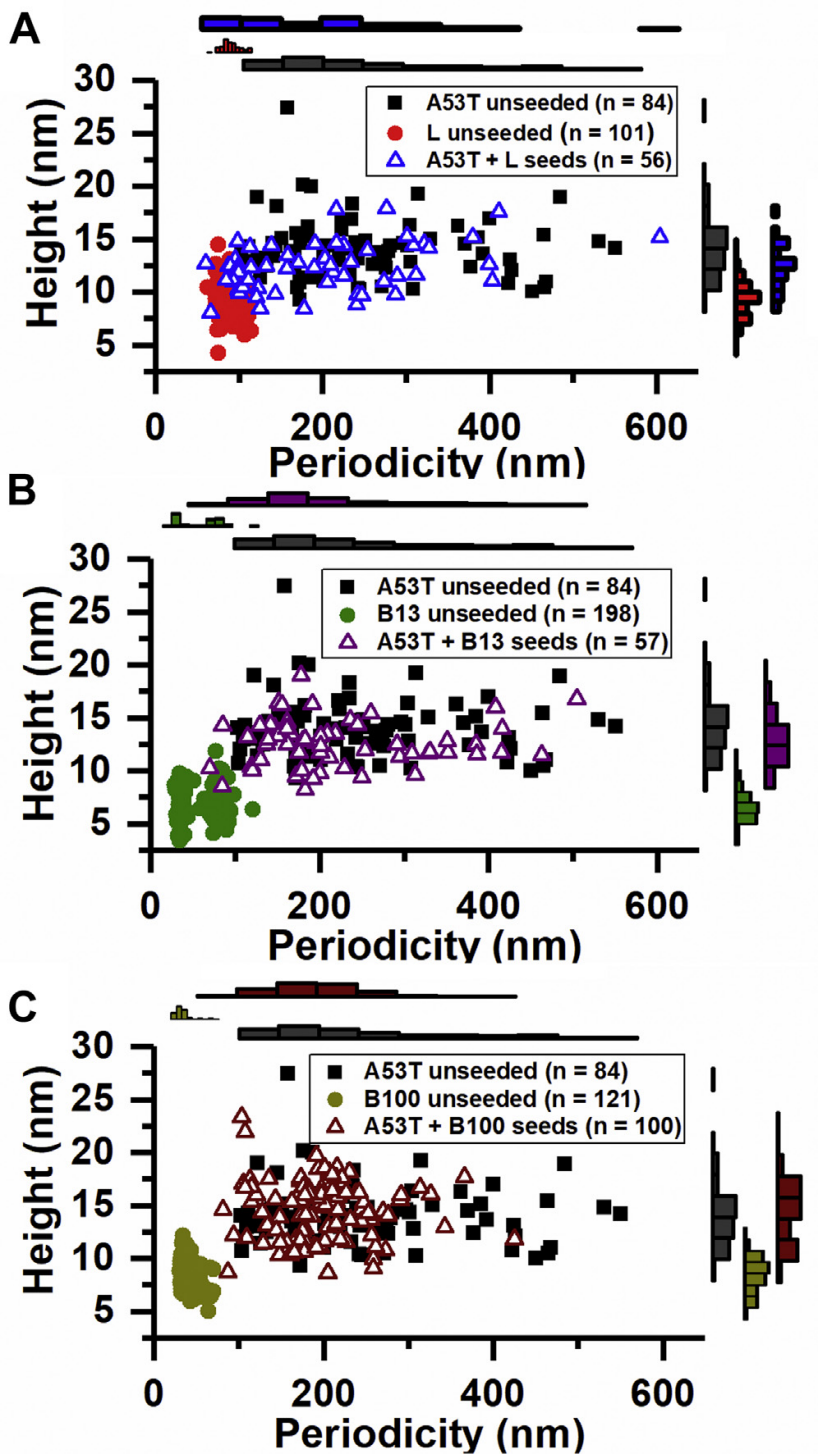
**aSyn A53T (98 μM) fibril morphology is not affected by cross-seeding with β-lactoglobulin or lysozyme fibril seeds (2 μM).** Height *versus* periodicity plots of cross-seeded A53T fibril populations for (*A*) lysozyme, (*B*) β-lactoglobulin polymorph B13, and (*C*) β-lactoglobulin polymorph B100 seeds. The histograms on top and next to the plots show the periodicity and height distributions. The n number in the legend is the number of fibrils analyzed.

Depending on the seeding mechanism, (1) seeds are incorporated in the fibril transferring the seed morphology to the growing aSyn fibril (seed elongation) (32). Alternatively, (2) the seed surface only acts as a catalyst for the nucleation of new aSyn fibrils (seed surface-mediated nucleation). In the latter case, the morphology of the fibril seed is not necessarily transferred. To investigate transmission of seed morphology to growing WT aSyn fibrils using AFM, differences in morphology of food protein fibrils and WT aSyn fibrils should be distinguishable. Preliminary AFM imaging of fibrils formed showed that the morphological characteristics of β-lactoglobulin, as characterized by fibril periodicity, and lysozyme fibrils largely overlap with those of WT aSyn fibrils (periodicity between 60–160 nm) (33). AFM analysis showed that the morphologies of lysozyme fibrils and E46K and A30P aSyn fibrils also overlap (Fig. S3). Only the morphologies of the lysozyme and A53T aSyn fibrils are sufficiently different to be distinguished in AFM experiments. The periodicity distribution of A53T aSyn fibrils is very disperse (100–550 nm) compared with the narrow distribution of lysozyme fibril periodicity (80–120 nm). Judging from the increase in ThT fluorescence with time, aggregation of A53T aSyn (Fig. S2*B*) in presence of lysozyme fibril seeds shows a similar trend to the results obtained with WT aSyn; the lag phase of A53T aSyn aggregation was significantly shorter in presence of lysozyme seeds. Hence, the transmission of lysozyme seed morphology was studied using A53T aSyn. To investigate if seeding by lysozyme seeds is accompanied by transmission of seed morphology to growing A53T fibrils, AFM images of heterologously seeded fibrils were recorded. Despite the finding that the lag phase of A53T aSyn fibril formation was significantly reduced by the presence of lysozyme, the morphological features of the formed A53T aSyn amyloid fibrils were not affected (Fig. 2) arguing for a seed surface-mediated nucleation mechanism.

**Figure 3:**
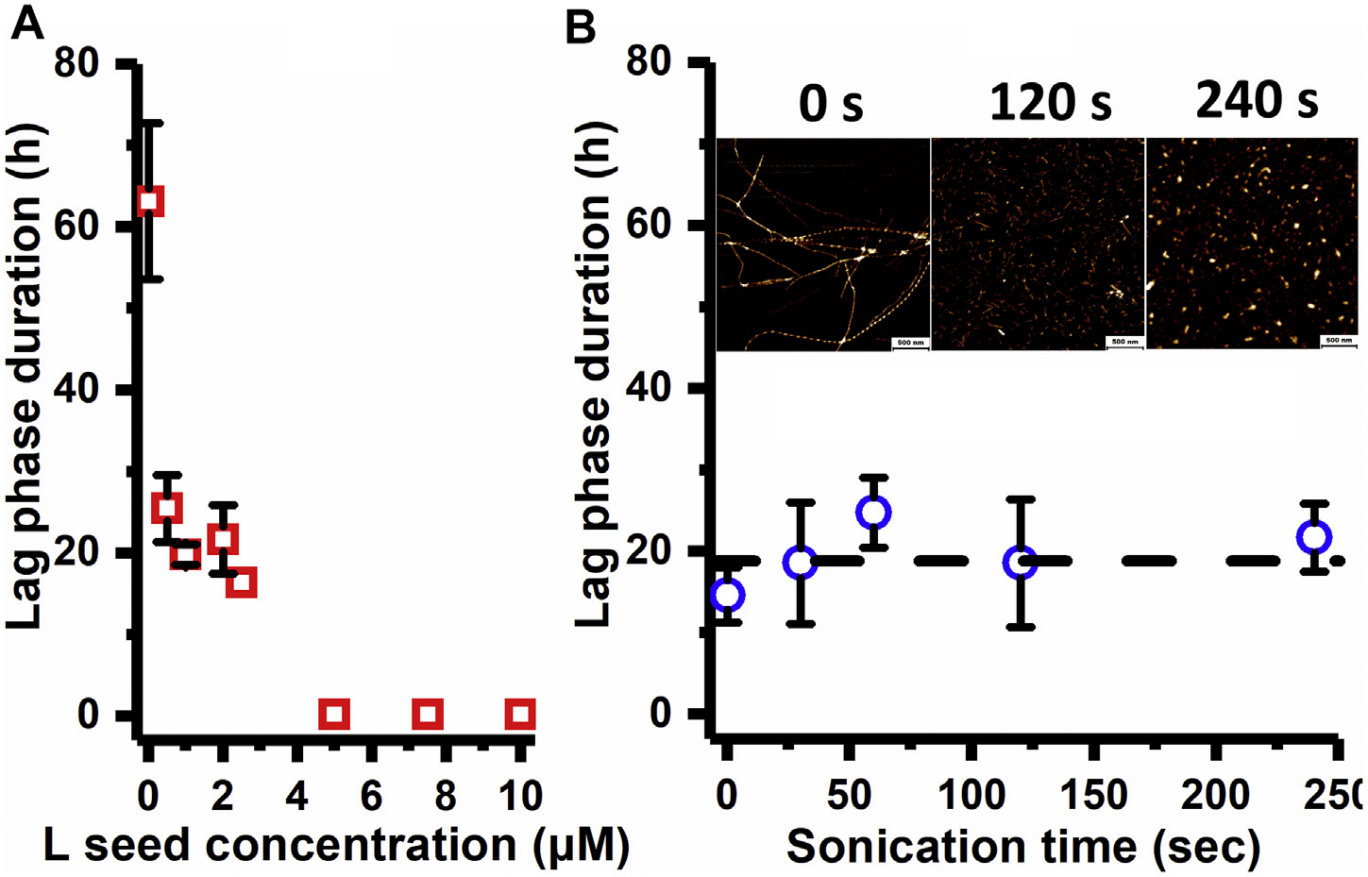
**A surface-mediated nucleation mechanism is responsible for the heterologous seeding of aSyn aggregation by lysozyme fibrils (“L”).***A*, reduction of the length of the lag phase as a function of the lysozyme seed concentration (equivalent monomer) for a fixed seed length distribution. *B*, length of the aSyn aggregation lag time at a fixed 2 μM lysozyme seed concentration as a function of the time, the seeds were subjected to sonication. With increasing sonication time, the lysozyme seeds become shorter as shown in the AFM images (0 s, 120 s and 240 s of sonication). Scale bar: 500 nm. The height scale is identical for the three images and varies from 0 (*black*) to 10 nm (*white*). Error bars denote standard deviations among triplicates. AFM, atomic force microscopy.

The lack of transfer of morphological characteristics of the lysozyme fibril seeds to growing aSyn fibrils hints toward a seed surface-mediated nucleation mechanism. Seeded fibril growth by seed elongation describes that monomeric protein is added at one or both extremities of the seeds. This type of seeding mechanism is characterized by a seeding efficiency that is directly proportional to the number of seed extremities present in the solution. Seed surface-mediated nucleation comprises the assembly of monomeric protein on the seed surface. In this case, the surface localized increase in monomer concentration induces the aggregation process, and the forming fibrils dissociate from the seed surface once a critical fibril mass has been formed. This type of seeding predicts that the seeding efficiency is directly proportional to the seed surface area available (32). To further discriminate which of the two seeding mechanisms dictates the cross-seeding effect of lysozyme seeds on aSyn aggregation, WT aSyn was aggregated in the presence of different lysozyme seed concentrations and followed in time using optical density (OD) measurements (Fig. S4). The lag phase of aSyn aggregation was found to decrease with increasing concentration of lysozyme seeds (Fig. 3*A*). In a second experiment, lysozyme seeds of various lengths while preserving total seed mass were prepared by exposing mature lysozyme fibrils to sonication for different amounts of time. When WT aSyn was aggregated in the presence of a fixed equivalent monomer concentration of lysozyme seeds (Fig. S4), the lag phase was not significantly affected by a change in sonication time (Fig. 3*B*). The efficiency by which lysozyme seeds enhance aSyn aggregation is thus dependent on available seed surface area and not on the number of seed ends.

**Figure 4:**
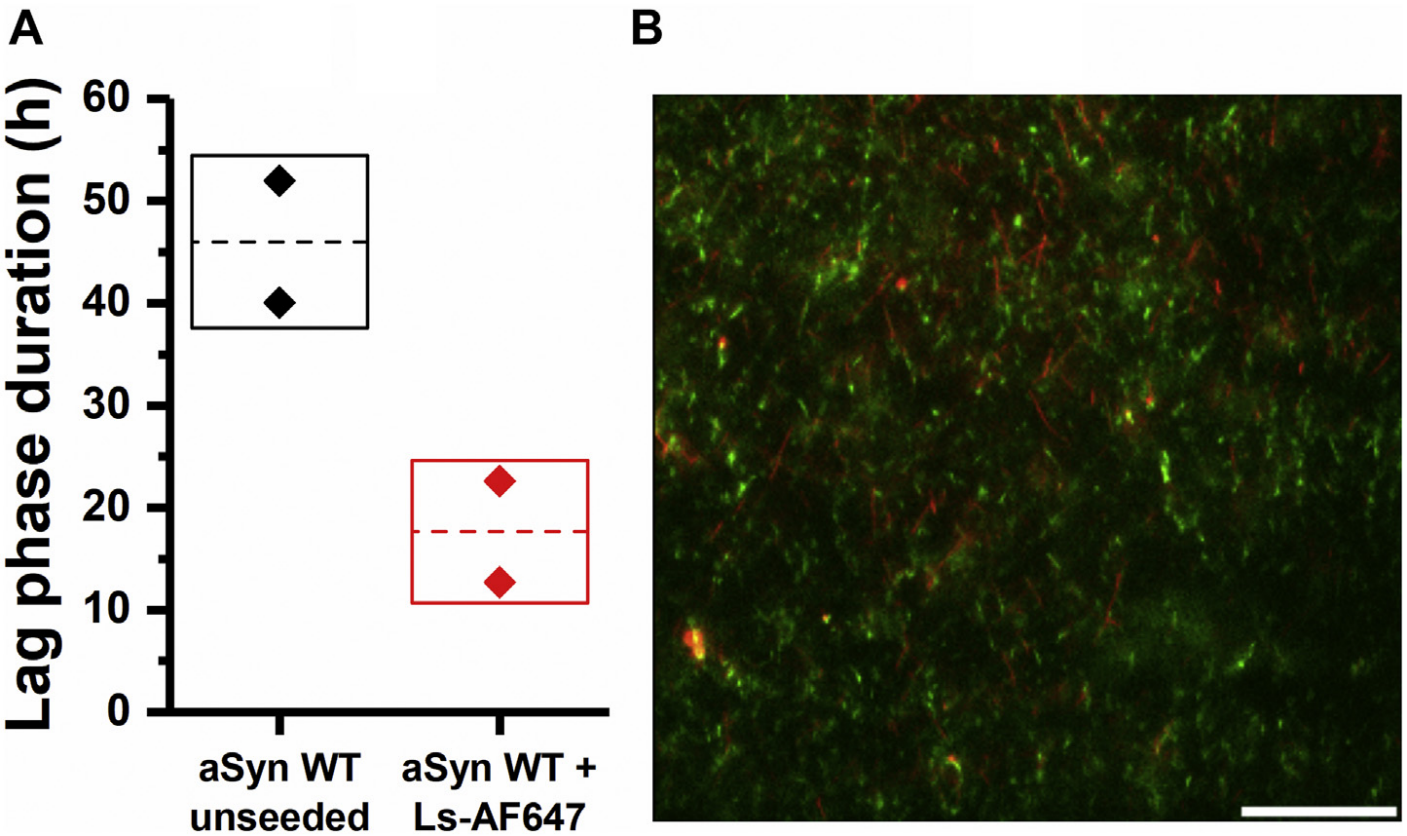
**Lysozyme seeds extremities are not extended by aSyn during the cross-seeding aggregation**. *A*, lag phase duration of 98 μM aSyn aggregation in absence (unseeded) or in presence of 2 μM labeled lysozyme-AF647 seeds (“Ls-AF647”) (2 μM). The mean and the standard deviations of the duplicates are represented by the *dashed line* and the *box*, respectively. *B*, representative fluorescence microscopy overview image of aSyn (ThT fluorescence—*green*) (98 μM) aggregated in the presence of lysozyme-AF647 (*red*) seeds (2 μM). Scale bar: 100 μm.

To exclude that the decrease in the aggregation lag time of aSyn in the presence of lysozyme seeds results from seed elongation, fluorescence microscopy experiments were performed. For this, AF488-labeled monomeric aSyn was aggregated in the presence of AF647-labeled lysozyme seeds under conditions where cross-seeding is observed (Fig. 4*A*). The resulting amyloid fibrils were visualized using fluorescence microscopy (Fig. 4*B*). Both green aSyn fibrils and red lysozyme fibrils can be individually observed without significant co-localization or overlap. The visual absence of green aSyn fibrils directly extending from red lysozyme fibrils indicates that aSyn monomers do not elongate from lysozyme seeds.

Collectively, these results show that the seeding effect of lysozyme seeds on aSyn aggregation is primarily characterized by a seed surface–mediated nucleation mechanism.

### Affinity between aSyn and amyloid food seeds

**Figure 5:**
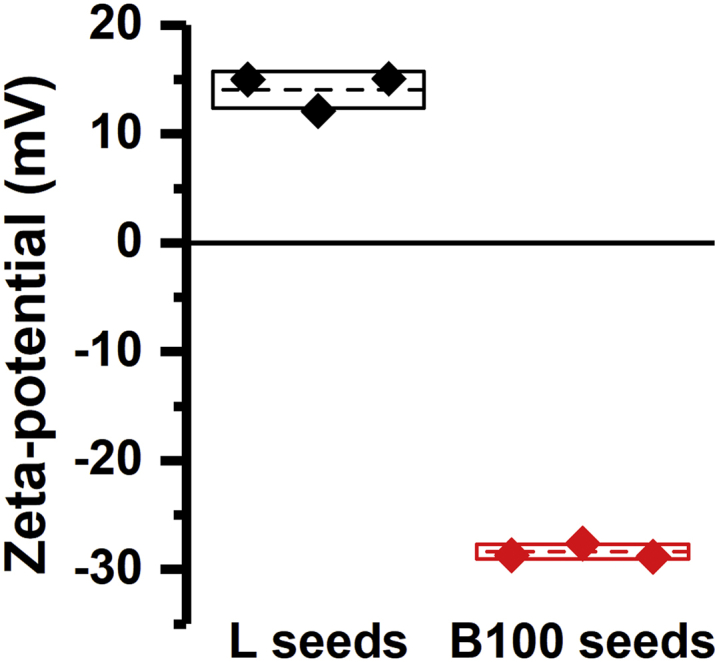
**Zeta-potential of lysozyme “L” and β-lactoglobulin “B100” seeds in aggregation buffer (10 mM Tris-HCl, 10 mM NaCl, 0.1 mM EDTA, pH 7.4)**. The mean and the standard deviation of the triplicates are represented by a *dashed line* and a *box*, respectively.

To explain why lysozyme seeds can cross-seed aSyn aggregation while β-lactoglobulin seeds lack this ability, we first considered the net charge of the seeds. Based on the pKa values of the amino acids, it is expected that aSyn and β-lactoglobulin are both net negatively charged at neutral pH, whereas lysozyme is net positively charged. To verify if information acquired on the basis of primary sequence translates into net oppositely charged amyloid fibril, we measured the zeta-potential of the seeds. As expected, while lysozyme seeds have a net positive surface charge, β-lactoglobulin polymorph B100 seeds have a net negative surface charge (Fig. 5). This observed difference in net surface charge potentially allows for attractive electrostatic interaction between lysozyme and aSyn. Such a mechanism would predict that lysozyme and β-lactoglobulin seeds show differential binding characteristics with aSyn. To investigate this, microscale thermophoresis (MST) experiments were performed. In these experiments, fluorescently labeled aSyn was titrated with lysozyme or β-lactoglobulin seeds. Binding curves were measured, and dissociation constants (K_D_) were derived from these curves. Because the seed surface–mediated nucleation process depends on the surface area and not on the particle concentration, this K_D_ is determined as an equivalent monomer concentration.

**Figure 6:**
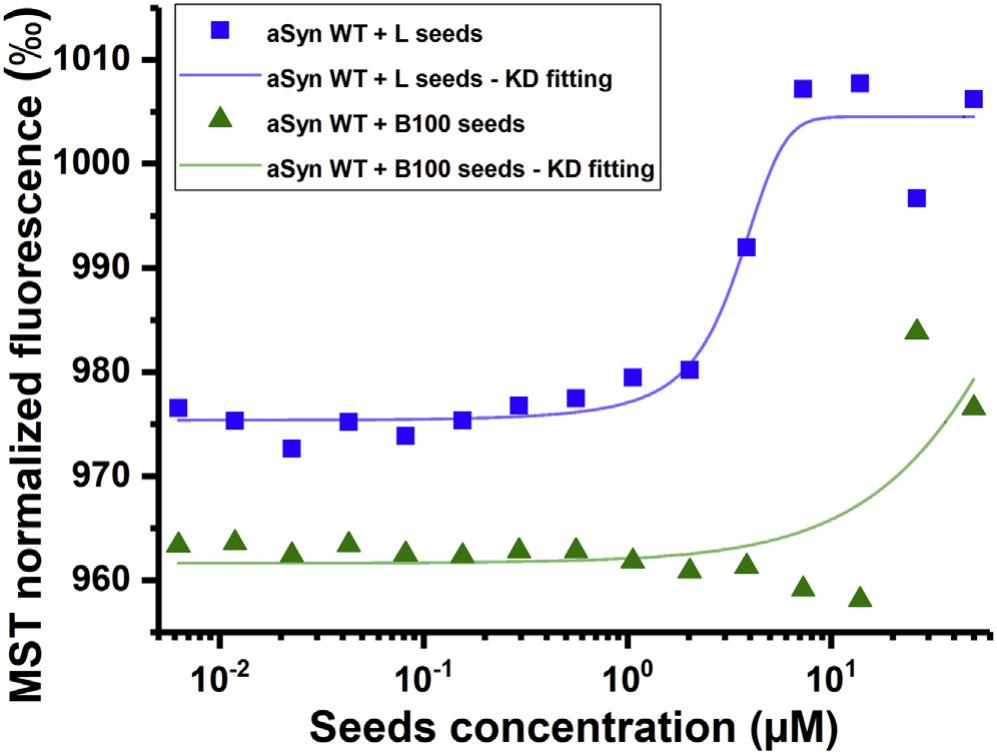
**aSyn interacts with higher affinity with lysozyme than with β-lactoglobulin seeds.** Example of binding curves for binding of WT aSyn monomers to β-lactoglobulin (“B100”, *green triangles*) and lysozyme (“L”, *blue squares*) seeds (in equivalent monomer concentrations) in 10 mM Tris-HCl, 0.1 mM EDTA, pH 7.4 buffer. *Colored lines* are the K_D_ fits of the data. MST, microscale thermophoresis.

From the MST binding curves (Fig. 6), a lower limit to the equilibrium dissociation constants was determined. The obtained K_D_ for the interaction with aSyn has a lower limit of 309 ± 34 μM for β-lactoglobulin seeds and 13 ± 11 μM for lysozyme seeds (based on triplicate measurements). Even though aSyn binds more strongly to lysozyme seeds than to β-lactoglobulin seeds, the micromolar binding affinities demonstrate a relatively weak interaction. In theory, such weak interactions may enable the assembly of surface-associated aSyn into energetically more favorable structures; strong binding would interfere with the structural rearrangements required for seed surface-mediated nucleation processes to take place. The observed order of magnitude difference in K_D_ between binding to lysozyme and β-lactoglobulin fibrils may not directly explain the difference in the ability to cross-seed aSyn aggregation but is consistent with a seed surface–mediated nucleation mechanism.

### aSyn region involved in the binding of amyloid food seeds

While the observed weak affinities of β-lactoglobulin and lysozyme seeds for aSyn binding do not sufficiently explain the difference in aSyn cross-seeding activity, it is possible that the aSyn regions involved in the interaction with the two types of seeds are different. As the aggregation prone region of aSyn has been identified as the NAC region (34), we anticipate that β-lactoglobulin and lysozyme seeds differentially affect exposure of this region to become available for aggregation.

To test this hypothesis, we employed an ELISA-based technique, based on the principle that if an aSyn region is involved in seed interaction, it is unavailable for interaction with a region-specific antibody. After coating of a 96-well plate with β-lactoglobulin or lysozyme seeds, monomeric WT aSyn was added to each well followed by incubation with one of three primary antibodies targeting different regions of aSyn: against the NAC region (epitope: aSyn 61–95), C-tail (epitope: aSyn 120–140), and full length aSyn (“FL”). As it was difficult to precisely quantify the amount of lysozyme or β-lactoglobulin seeds coated in each well, per type of aSyn-seed complex the resulting chemiluminescence signals of the C-tail antibody experiments were divided by either the signal intensities obtained using the NAC or FL targeting antibodies.

**Figure 7:**
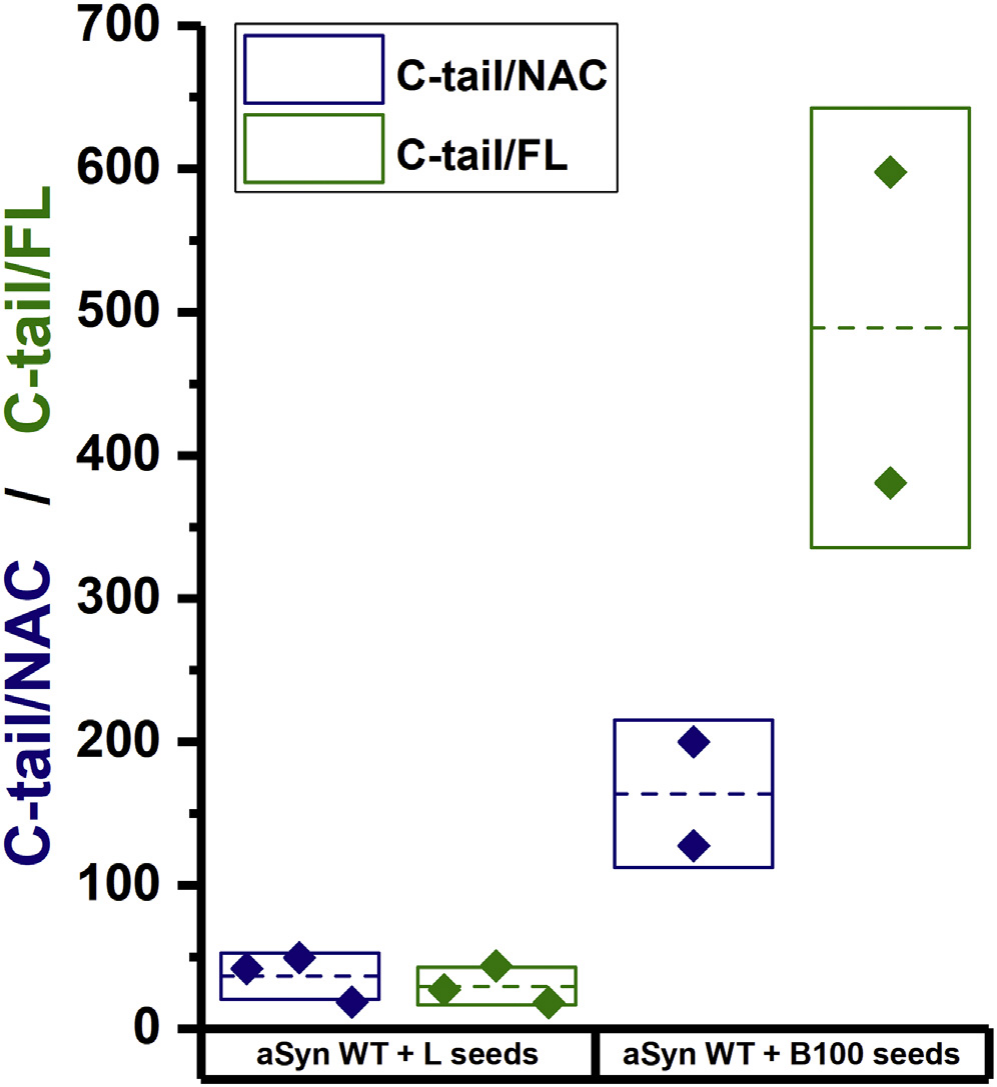
**Seeds derived from lysozyme and β-lactoglobulin fibrils interact with distinct regions of aSyn**. ELISA assay to identify interaction of lysozyme (“L”) or β-lactoglobulin polymorph 100 (“B100”) seeds with different regions of aSyn using antibodies against the NAC region (NAC), C-tail (C-tail), or full length aSyn (FL). The chemiluminescence signals of the C-tail antibody experiments were divided by either the signal intensities obtained using the NAC (C-tail/NAC; *blue*) or FL targeting antibodies (C-tail/FL; *green*). The mean and the standard deviation of the duplicates/triplicates are represented by a *dashed line* and a *box*, respectively.

Even though based on this assay the specific region with which the seeds interact cannot be identified, comparison of resulting C-tail/NAC ratios (Fig. 7) suggests that a large difference exists in the availability of these regions upon incubation of aSyn with seeds derived from either lysozyme or β-lactoglobulin. The C-tail/NAC ratio is higher when aSyn is incubated with β-lactoglobulin seeds compared with when aSyn is incubated with lysozyme seeds. These results imply that the C-tail of aSyn is relatively exposed in the presence of β-lactoglobulin seeds compared with lysozyme seeds. At the same time, the NAC region interacts in binding, albeit at low affinity (as observed from MST), with β-lactoglobulin but not lysozyme seeds. The interaction between aSyn C-tail and lysozyme seeds should decrease the long-range repulsion between aSyn monomers and thus promote aggregation.

**Figure 8:**
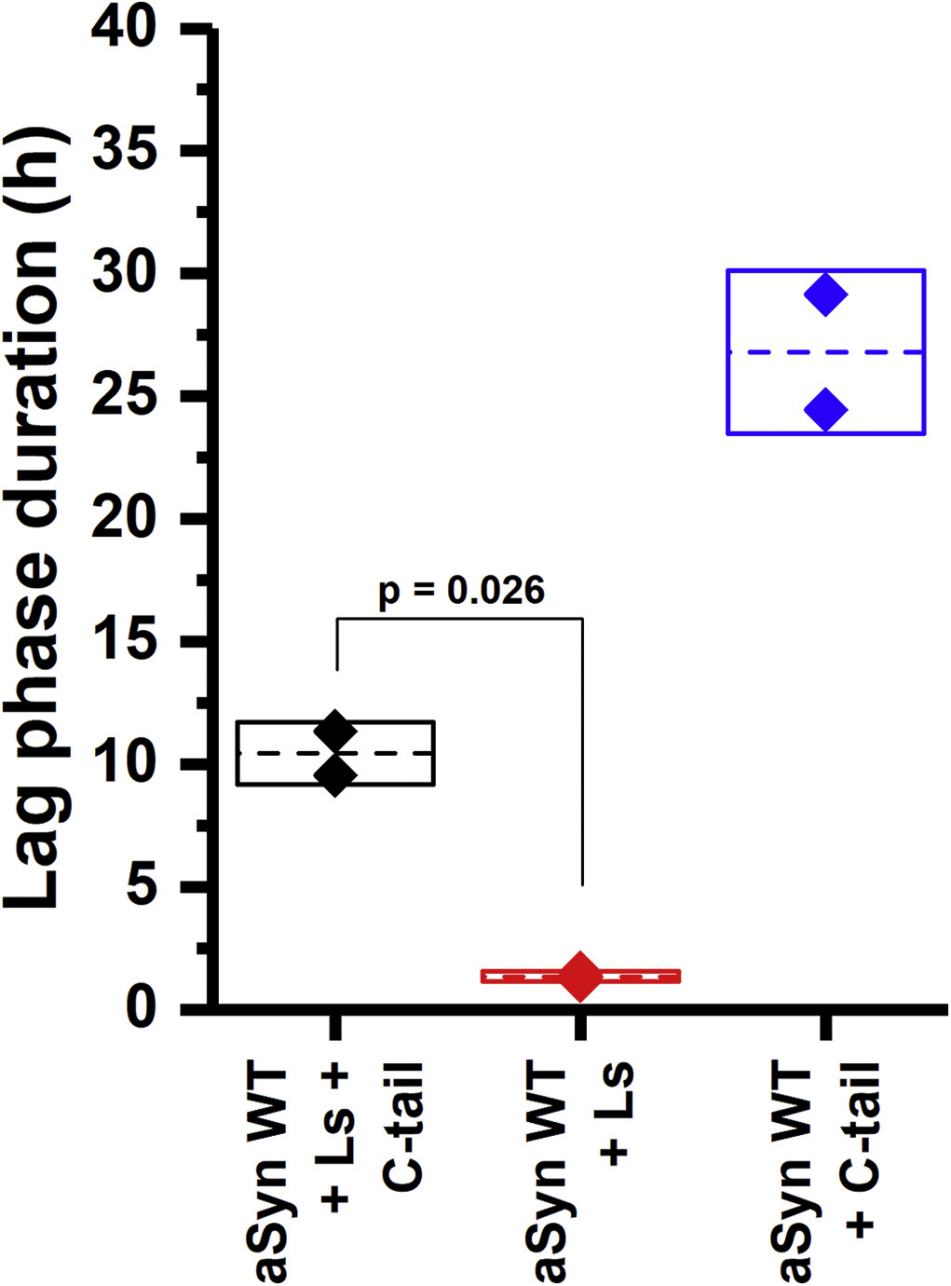
**Aggregation in the presence of aSyn C-tail peptide (“C-tail”) reduces the effect of lysozyme seeds (“Ls”) on the aSyn WT aggregation lag phase**. The mean and the standard deviations of the duplicates are represented by the *dashed line* and *the box*, respectively. The *p*-value is calculated based on a t-test.

To support our hypothesis that the interaction of the C-terminus of aSyn with the lysozyme seed is important for cross seeding to occur, a competitive cross-seeding experiment was performed. In this experiment, a C-terminal aSyn peptide comprising of amino acids 96 to 140 and full-length WT aSyn competed for cross-seeding with the lysozyme seeds in an aggregation experiment. The presence of the C-terminal peptide significantly elongated (*p*-value = 0.026) the aggregation lag time (Fig. 8). The increase in the lag time indicates that aSyn C-tail peptide competes with WT aSyn binding to lysozyme seeds. The occupation of the binding sites on the lysozyme surface by the aSyn C-tail peptide decrease the ability of lysozyme seeds to catalyze the aggregation of WT aSyn. This agrees with the expected role of charge–charge interactions between the net negatively charged aSyn C-tail and the net positively charged lysozyme surface in mediating the interaction.

## Discussion

The clinically observed co-occurrence of various protein aggregation–associated pathologies is poorly understood. For example, Lewy body-pathology is frequently observed in Alzheimer's disease suggesting that aggregation of aSyn co-occurs with the characteristic precipitation of Alzheimer's disease proteins Aβ peptide and tau (35, 36). Similarly, huntingtin, the protein implicated in Huntington's disease, can be found co-localized with intracellular tau lesions in Alzheimer's disease and cytoplasmic Pick bodies in Picks disease (37). Until now, it has been unclear how these observations can be reconciled, but the idea that amyloid fibrils of one protein species can, in some cases, induce the aggregation of other proteins is further supported by *in vitro* studies which confirm that aSyn aggregation can be cross-seeded by Aβ amyloid fibrils (9).

In our test tube study, we show that fibril cross-seeding of aSyn aggregation by heterologous proteins can occur. The nucleation of aSyn fibrils is catalyzed by the presence of lysozyme fibrils in which the lysozyme fibril surface acts as a catalyst. In solution, the C-terminal region of aSyn protects aSyn against aggregation by its net negative charge density and intramolecular interactions with the N-terminal region which screen the amyloidogenic aSyn NAC region and prevent it from aggregation (38). Based on our results, we hypothesize that lysozyme seeds interact with the aSyn C-terminal region resulting in less efficient screening of the amyloidogenic aSyn NAC region, thus leaving aSyn in a more aggregation prone conformation on the lysozyme fibril surface.

The observation that lysozyme effectively cross-seeds aggregation of aSyn in our test tube assays raised the question if aSyn and lysozyme amyloid fibrils can come into direct physical contact *in vivo*. Peripheral (aggregated) sources of aSyn protein were detected in the intestine and were shown to induce brain neurodegeneration (39). The trafficking of pathological forms of aSyn from the intestine to the brain may involve vagus nerve–mediated transport, among multiple other identified routes (40, 41, 42). The vagus nerve, *via* the enteric nervous system, directly innervates the intestine regulating intestinal locomotor activity, permeability, and bacterial homeostasis. Interestingly, nonmotor gastrointestinal clinical symptoms such as constipation, dysbiosis, and leaky gut are commonly observed in patients suffering from PD several years before onset of motor-related clinical symptoms (43). A recent study in a mouse model showed that gastrointestinal aSyn fibrils can reach the brainstem by means of vagus nerve–mediated transport as vagus nerve severing ameliorated early PD-like pathogenesis (41). Eggs are a rich source of lysozyme, and processing of eggs before ingestion, by means of baking or boiling, is known to induce aggregation of lysozyme into amyloid fibrils (44). Hen egg white lysozyme is resistant against digestion in the gastric and duodenal compartments of the intestine and demonstrates residual enzymatic activity following exposure to digesting conditions (45). While it is conceivable that ingested lysozyme seeds in the gastrointestinal tract cross-seed gastrointestinal aSyn, alternatively lysozyme amyloid fibrils may enter the brain *via* the vagus nerve. Collectively, lysozymal digestic resistance, detection of gastrointestinal aSyn aggregates, PD-associated gastrointestinal symptoms, and the vagus nerve–mediated transport of aSyn toward critical PD-affected brain regions highlights the possibility that lysozyme fibrils and aSyn can physically interact in the brain.

Our results indicate that amyloid proteins from external sources, like food products, can potentially induce aggregation of disease-related brain-residing proteins. However, such heterologous cross-seeding effect may be specific as β-lactoglobulin was shown to lack the capacity to induce aSyn aggregation.

In this work, we investigated the ability of amyloid fibrils derived from two food proteins to heterologously seed the aggregation of the PD-related aSyn protein. While β-lactoglobulin amyloid fibril seeds were not able to induce aSyn aggregation, we identified a fibril surface–mediated nucleation mechanism for the seeding of aSyn aggregation by hen egg white lysozyme fibrils. We could attribute this difference in seeding capacity to differences in the affinity of aSyn for both fibril surfaces and aSyn region responsible for this surface binding.

## Experimental procedures

### Materials

Hen egg white lysozyme (cat. no. L6876) and bovine milk β-lactoglobulin (cat. no. L3908) were obtained from Sigma and used without further purification.

### Expression and purification of aSyn WT and disease mutants

WT, A140C, and disease-related mutant (A30P, E46K, and A53T) aSyn were produced recombinantly as described previously (46). Briefly, proteins were produced in *Escherichia coli* cells transformed with the pT7-7 plasmid carrying the WT or a modified aSyn gene. After induction by isopropyl β-d-1-thiogalactopyranoside, *E.coli* cells were lysed, and aSyn proteins were purified by standard methods, including ion-exchange chromatography. Aliquots were stored at −80 °C.

### Aggregation of food proteins

#### Lysozyme aggregation

A lysozyme solution was prepared by dissolving 1.43 mg lyophilized lysozyme powder in 50 mM NaH_2_PO_4_, pH 2.0 and subsequent filtering through a 0.02 μm pore size syringe filter (Anotop 10; Whatman GE Healthcare). After determining the protein concentration by measuring the absorbance of the solution at 280 nm (Thermo Scientific NanoDrop TM 1000 Spectrophotometer) and using a molar extinction coefficient of 36,000 M^−1^ cm^−1^, the lysozyme solution was diluted to a concentration of 100 μM. All aggregation reactions were prepared in triplicate with a final volume of 400 μl in 2 ml LoBind round bottom Eppendorf centrifuge tubes. The solutions were incubated at 80 °C in an Eppendorf Thermomixer Comfort at an orbital shaking speed of 750 rpm for 25 h. A ThT binding assay was used as read-out for aggregation.

#### *β*-Lactoglobulin aggregation

To produce two different structural polymorphs of β-lactoglobulin amyloid fibrils, 1.84 mg β-lactoglobulin powder was dissolved in 13 mM or 100 mM NaCl, pH 2.0 and subsequently filtered through a 0.02 μm pore size syringe filter (Anotop 10; Whatman GE Healthcare). After determining the protein concentration by measuring the absorbance of the solution at a wavelength of 280 nm (Thermo Scientific NanoDrop TM 1000 Spectrophotometer) and using a molar extinction coefficient of 17,600 M^−1^ cm^−1^, the β-lactoglobulin solutions were diluted to a concentration of 100 μM. Each aggregation reaction was prepared in triplicate with a final volume of 400 μl in 2 ml LoBind round bottom Eppendorf centrifuge tubes. The solutions were incubated at 80 °C in an Eppendorf Thermomixer Comfort at an orbital shaking speed of 750 rpm. A ThT binding assay was used as read-out for aggregation. The resulting samples in 13 mM NaCl and 100 mM NaCl were named “B13” and “B100”, respectively.

### Unseeded aSyn aggregation

Aliquots of WT and disease mutant aSyn were thawed and filtered through a 0.02 μm pore size filter (Anotop 10; Whatman, GE Healthcare). Aggregation reactions were performed with 100 μM aSyn in 10 mM Tris-HCl, 10 mM NaCl, 0.1 mM EDTA, pH 7.4 (buffer A). Each aggregation reaction was prepared in triplicate at a final volume of 400 μl in 2 ml LoBind round bottom Eppendorf centrifuge tubes. The solutions were incubated at 37 °C in an Eppendorf Thermomixer Comfort at an orbital shaking speed of 750 rpm, and a ThT fluorescence assay was used as read-out for aggregation.

### Heterologously seeded aSyn aggregation

Aliquots of WT or disease mutant aSyn were thawed and filtered through a 0.02 μm pore size syringe filter (Anotop 10; Whatman, GE Healthcare). Monomeric aSyn solutions were prepared at 98 μM in buffer A, and 2 μM monomer equivalent amyloid food seeds (see below) were added. Each aggregation reaction was prepared in triplicate at a final volume of 400 μl in 2 ml LoBind round bottom Eppendorf centrifuge tubes. The samples were incubated at 37 °C in an Eppendorf Thermomixer Comfort at an orbital shaking speed of 750 rpm, and a ThT fluorescence assay was used as read-out for aggregation.

### Aggregation reactions in multiwell plates

For well plate measurements, similar aggregation reactions as described above were prepared in triplicate in 96-well plates (black-walled, transparent, flat bottom, Greiner) at a final volume of 200 μl/well.

For the competitive cross-seeding of aSyn aggregation, a peptide fragment consisting of amino acids 96 to 140 of aSyn (aSyn C-tail peptide—rPeptide S-1014-1) was added to the aggregation reaction at a final concentration of 50 μM.

The plates were sealed with a transparent plastic sheet and incubated at 37 °C in a Tecan Safire2 plate reader (or Tecan Infinite pro 200) under orbital shaking at 430 rpm. The aggregation kinetics was followed by measuring the OD at a wavelength of 340 nm (OD_340_) with a time resolution of 15 min instead of using ThT fluorescence to prevent any cross-interaction from ThT.

### Amyloid seed preparation

Amyloid seeds were prepared by sonication of 50 to 100 μl of a 100 μM amyloid fibril preparation in 200 μl thin-walled PCR tubes for 4 min in a bath sonicator (Branson 2510) at room temperature. The fragmentation efficiency of the sonication was evaluated by AFM imaging (described later) of the resulting seed solution.

### Thioflavin-T assay

A ThT solution was prepared at about 1 mM in 50 mM Glycine-NaOH, pH 8.2 buffer. After filtering through a 0.2 μm pore size filter, the ThT concentration was calculated by measuring the absorbance at a wavelength of 412 nm and using an extinction coefficient of 26,620 M^−1^ cm^−1^. The ThT stock solution was stored in aliquots at −20 °C. Before use, a 5 μM working solution was freshly prepared by dilution in 50 mM Glycine-NaOH, pH 8.2. At different incubation time points, 5 μl aliquots of the aggregation reactions were diluted in 2 ml of 5 μM ThT and incubated for 5 min at room temperature. The fluorescence intensity of the mixture was measured using a Cary Eclipse Fluorescence spectrophotometer (Varian Inc). Emission spectra were recorded from 475 to 600 nm with excitation at 458 nm, using excitation/emission slit widths of 10 nm. Aggregation kinetic curves were obtained by plotting the emission fluorescence at 485 nm *versus* time.

### Lag phase determination

The aggregation of aSyn into β-sheet–rich structures as a function of time was fitted to a sigmoid (Boltzmann fitting):

$$y=A_{2}+\frac{A_{1}-A_{2}}{1+e^{\frac{\left(x-x_{0}\right)}{dx}}}$$

where A_1_ and A_2_ are the initial and the final fluorescence intensity/OD values, respectively; x_0_ is the time when fluorescence/OD reaches the half maximum value, and dx is the inverse of the elongation rate constant. By fitting the different parameters were determined. The lag time was calculated with this equation (47):

$$lag\;time=x_{0}-2dx$$

### Atomic force microscopy

A volume of 20 μl of five to ten times diluted fibrils (initial concentration ≈ 100 μM (monomer based)) in buffer A was adsorbed onto freshly cleaved mica (Muscovite mica, V-1 quality, EMS) for 4 min. The sample on mica was carefully washed three times with 100 μl of MilliQ water and dried under a gentle stream of a N_2_ gas. AFM images were acquired on a Bioscope Catalyst (Bruker) in soft tapping mode using a silicon probe, NSC36 tip B with a force constant of 1.75 N/m (MikroMasch). All images were captured with a resolution of 512 × 512 pixels per image at a scan rate of 0.5 to 1 Hz. AFM images were qualitatively analyzed with Scanning Probe Image Processor-6.0.13 (SPIP; Image Metrology) software. For quantitative analysis of fibril morphology Matlab R2016b software was used in combination with DIPimage toolbox (version 2.3) and a custom written fibril analysis script (48) to determine average fibril height and periodicity (pitch size of twisted fibril).

### Zeta-potential measurement

A 1 ml volume of 100 μM lysozyme or β-lactoglobulin polymorph 100 amyloid seeds were centrifuged at 21,000*g* for 1 h at room temperature. The pellet was resuspended in 1 ml of buffer A. The resulting amyloid seed solutions were injected in a DTS1060 cell using a 1 ml syringe, and the Zeta potential was measured using a ZetaSizer Nano ZS (Malvern Instruments). Each measurement was performed three times from the same amyloid seed solution.

### Protein labeling

Maleimide functionalized Alexa Fluor (AF) dye was used to label aSyn A140C monomers. A quantity of 1 mg of maleimide functionalized AF488/AF647 was dissolved in dry dimethyl sulfoxide to obtain a 25 mM stock solution. To reduce potential intermolecular disulfide bonds, 1 mM Tris(2-carboxyethyl)phosphine was added to 200 μl of 100 μM aSyn A140C (in buffer A) and incubated for 30 min at room temperature. Next, maleimide AF488/AF647 was added to the reduced aSyn monomers to a final dye concentration of 300 μM. The mixture was incubated in the dark for 1 h at room temperature, followed by removal of excess dye by two sequential desalting steps using 2 ml Zeba Spin 7K molecular weight cut-off desalting columns. The final protein concentration was calculated by measuring the absorbance at wavelengths of 280 nm and 493/650 nm and using the following equations:

$$C_{protein}=\frac{A_{280}-CF\cdot A_{493/650}}{\varepsilon_{protein}}$$

where A_280_ and A_493/650_ are the absorbance at 280 nm and 493/650 nm, respectively; ε_protein_ and ε_dye_ are the molar extinction coefficients of the aSyn protein at 280 nm (ε_A140C, 280 nm_ = 5747 M^−1^ cm^−1^), of AF488 dye at 493 nm (ε_AF488, 493 nm_ = 72,000 M^−1^ cm^−1^), and of AF647 dye at 650 nm (ε_AF647, 650 nm_ = 239,000 M^−1^ cm^−1^), respectively, and CF is the correction factor for the contribution to the A_280_ absorbance of the AF488 dye (CF_AF488_ = 0.11) or AF647 dye (CF_AF647_ = 0.05). Labeled protein solutions were stored as 20 μl aliquots at −20 °C.

### Microscale thermophoresis

The possible binding between aSyn and amyloid food seeds was determined using a Monolith NT.115 (NanoTemper Technologies GmbH) MST system equipped with a blue-green laser. The thermophoretic movement was monitored at a constant 50 nM concentration of A140C-AF488, combined with a dilution series of 16 different concentrations of amyloid seeds, ranging from 0 to 100 μM (equivalent monomer concentration) and prepared in 10 mM Tris-HCl, 10 or 50 mM NaCl, 0.1 mM EDTA, pH 7.4. Samples were transferred to capillaries (Standard treated, NanoTemper Technologies GmbH), and measurements were performed at 37 °C with a constant blue LED power of 20% and at three different MST infrared laser powers (20–40–80%) for preparation of a temperature gradient to induce thermophoretic movement. For each capillary, the measurements were performed as follows: a 5 s baseline for signal normalization, then a 30 s period with the infrared laser turned on, followed by another 5 s after turning off the laser to record the back diffusion of fluorescently labeled aSyn molecules. For evaluation, the normalized fluorescence intensity signal was plotted against the titrated seed concentration (equivalent monomer concentration). Data were analyzed with the MO.Affinity Analysis v2.1.2030 software using the Thermophoresis with T Jump strategy. Results were fitted to obtain an equilibrium dissociation constant K_D_ (MO.Affinity—standard fitting mode derived from the law of mass action).

### Fluorescence microscopy

Monomeric lysozyme was labeled with AF647 as described previously in the Protein labeling section.

Labeled lysozyme-AF647 fibrils were prepared by incubation of 90 μM unlabeled lysozyme monomers, 8 μM lysozyme seeds, and 2 μM labeled lysozyme-AF647 monomers in conditions where lysozyme aggregates (incubation at 80 °C in an Eppendorf Thermomixer Comfort at an orbital shaking speed of 750 rpm for 25 h).

Monomeric aSyn (98 μM) was incubated with labeled lysozyme-AF647 seeds (2 μM) at 37 °C in an Eppendorf Thermomixer Comfort at an orbital shaking speed of 750 rpm. After 2 days of incubation, fibrils were diluted 10 times in 20 μM ThT solution. A volume of 5 μl sample was placed on a microscope slide and covered by a coverslip. The edges were sealed with nail polish.

Fluorescence images were acquired in wide-field mode using an Olympus Plan Apo oil immersion objective with 100× magnification (NA = 1.45) on a Nikon Eclipse Ti microscope equipped with a mercury arc lamp (Nikon Intensilight, C-HGFIE) for excitation. For imaging of ThT fluorescence, a filter cube composed of a 445/465 nm band-pass excitation filter, a 458 nm long pass dichroic beam splitter, and a 470/500 nm band-pass emission filter were used. For imaging of AF647 fluorescence, a filter cube consisting of a 590/650 nm band-pass excitation filter, a 660 nm long pass dichroic beam splitter, and a 662/737 nm band-pass emission filter was used.

### Epitope mapping

Wells of a 96-wells plate (white plate, flat bottom, Greiner) were incubated with 100 μl of 1 μM monomer equivalent lysozyme seeds, β-lactoglobulin seeds or WT aSyn monomers in buffer A for 1 h at 37 °C. Subsequently, each well was washed three times with 200 μl of buffer A containing 0.05% Tween 20. To avoid nonspecific interactions, wells were subsequently incubated with 300 μl of 2.5% (w/v) BSA in buffer A. Then, each well was washed three times with 200 μl of buffer A containing 0.05% Tween 20. Next, wells with food protein seeds were incubated with 100 μl of 100 μM WT aSyn for 1 h at 37 °C. Subsequently, each well was washed three times with 200 μl of buffer A containing 0.05% Tween 20. All wells were incubated with one of the following primary antibodies: (i) Mouse anti-aSyn 61 to 95 (NAC region), named “NAC” (Enzo Life Sciences—ALX-804-656), diluted 1:500, (ii) Mouse anti-aSyn 15 to 123, named “FL” (BD science—610786), diluted 1:250, and (iii) Rabbit polyclonal anti-C-tail of aSyn, named “C-tail” (Santa Cruz—sc-7011-R), diluted 1:100 for 1 h at 37 °C. Then, each well was washed three times with 200 μl of buffer A containing 0.05% Tween 20. Wells were then incubated with one of the following secondary antibodies: (i) Goat anti-mouse HRP-conjugated (Sigma—A9917), diluted 1:60,000 or (ii) Goat anti-rabbit HRP-conjugated (Invitrogen—G21234), diluted 1:20,000 for 1 h at 37 °C. Subsequently, each well was washed three times with 200 μl of buffer A containing 0.05% Tween 20. Chemiluminescence was induced by addition of 100 μl of a H_2_O_2_/luminol (1:1) solution (Thermo Scientific Super Signal West Pico Chemiluminescent Substrate kit) to each well. Chemiluminescence was recorded using a Tecan Infinite Pro 200 plate reader with an integration time of 500 ms, and the signal after 40 min was used for data analysis.

## Data availability

All remaining data are contained within the article.

## Conflict of interest

The authors declare that they have no conflicts of interest with the contents of this article.
