# Supplementary material for: Cross-seeding of alpha-synuclein aggregation by amyloid fibrils of food proteins
Source: J Biol Chem. 2021 Feb 2;296:100358. doi: 10.1016/j.jbc.2021.100358 (PMC7949133; doi:10.1016/j.jbc.2021.100358)
Supplement: Supplementary file 1 — Figures S1 to S4 [file mmc1.pdf]

Cross-seeding of alpha-synuclein aggregation by amyloid fibrils of food proteins

Jonathan Vaneyck [1*], Ine Segers-Nolten[1], Kerensa Broersen[2] and Mireille M.A.E. Claessens[1]

[1]Nanobiophysics, MESA+ Institute for Nanotechnology, University of Twente, P.O. box 217, 7500 AE, Enschede, The Netherlands
[2]Applied Stem Cell Technologies, Technical Medical Centre, University of Twente, P.O. Box 217, 7500 AE, Enschede, The Netherlands

Supporting Information

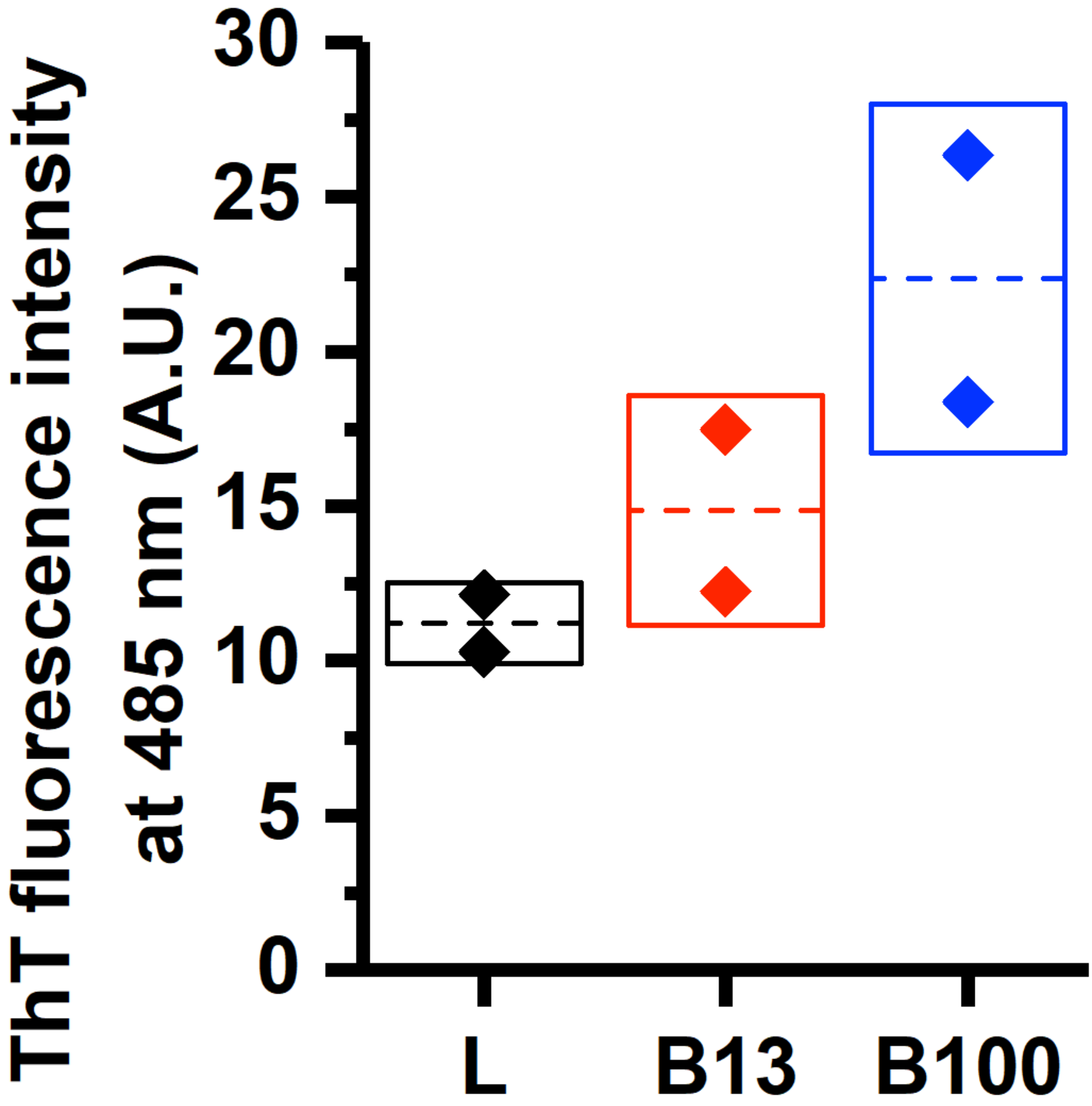

Figure S1: Lysozyme and β-lactoglobulin aggregates are ThT-positive aggregates. Final ThT fluorescence intensities at 485 nm are shown after 25 h of aggregation of 100 µM lysozyme ('L'), β-lactoglobulin polymorph B13 ('B13') and polymorph B100 ('B100'). The mean and the standard deviation of the duplicate represented by the dashed line and the box, respectively.

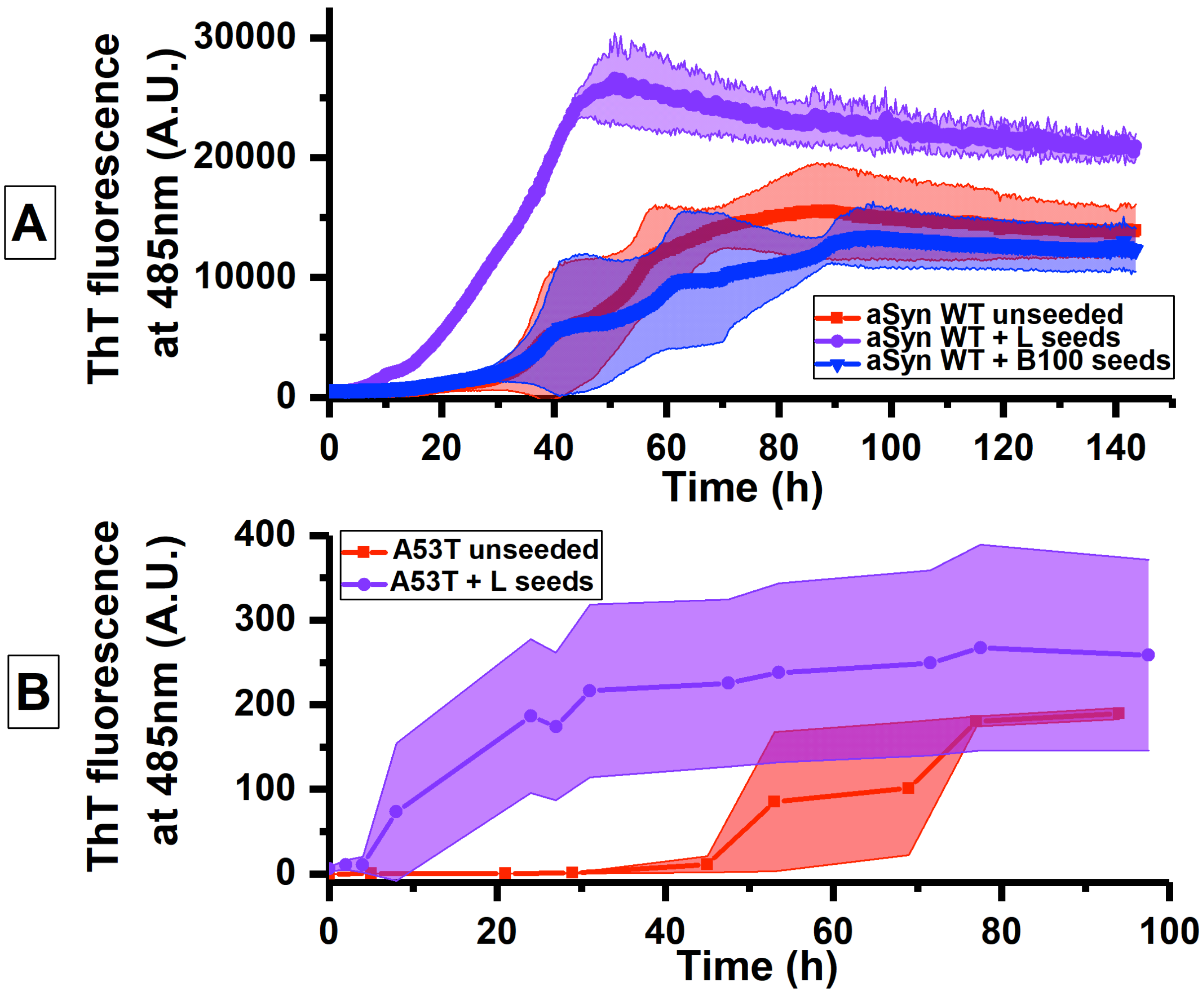

Figure S2: WT and A53T aSyn aggregation are similarly nucleated by the presence of lysozyme seeds. A) WT and B) A53T aSyn aggregation curves in absence ('unseeded') or in presence of lysozyme ('L') or β-lactoglobulin polymorph B100 ('B100') seeds by following ThT fluorescence over time. Shaded areas correspond to error bars (which denote standard deviations of triplicate measurements).

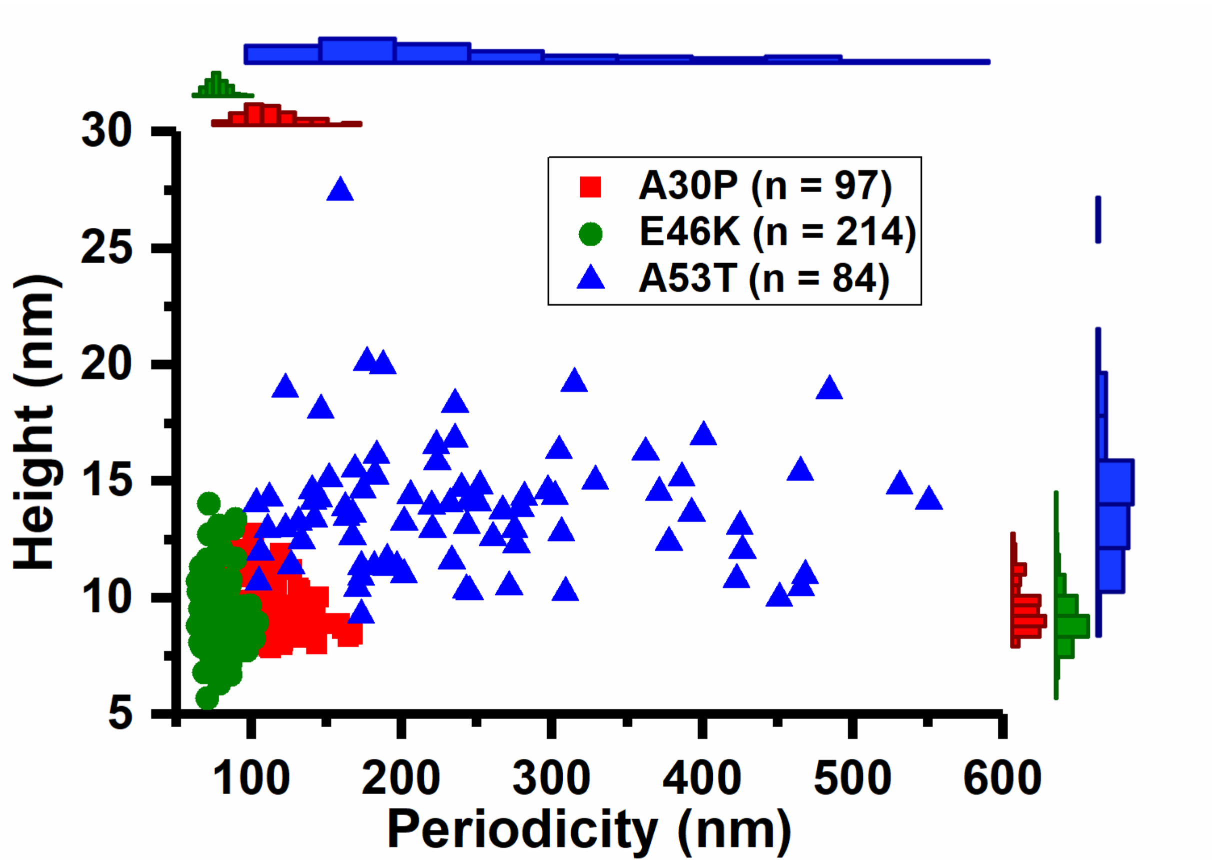

Figure S3: Fibrils of disease-related aSyn mutants show different morphological characteristics. Height versus periodicity plot based on AFM image analysis of A30P (red), E46K (green), and A53T (blue) fibrils. The histograms on top and next to the plots show the periodicity and height distributions. The n number in the legend is the number of fibrils analyzed.

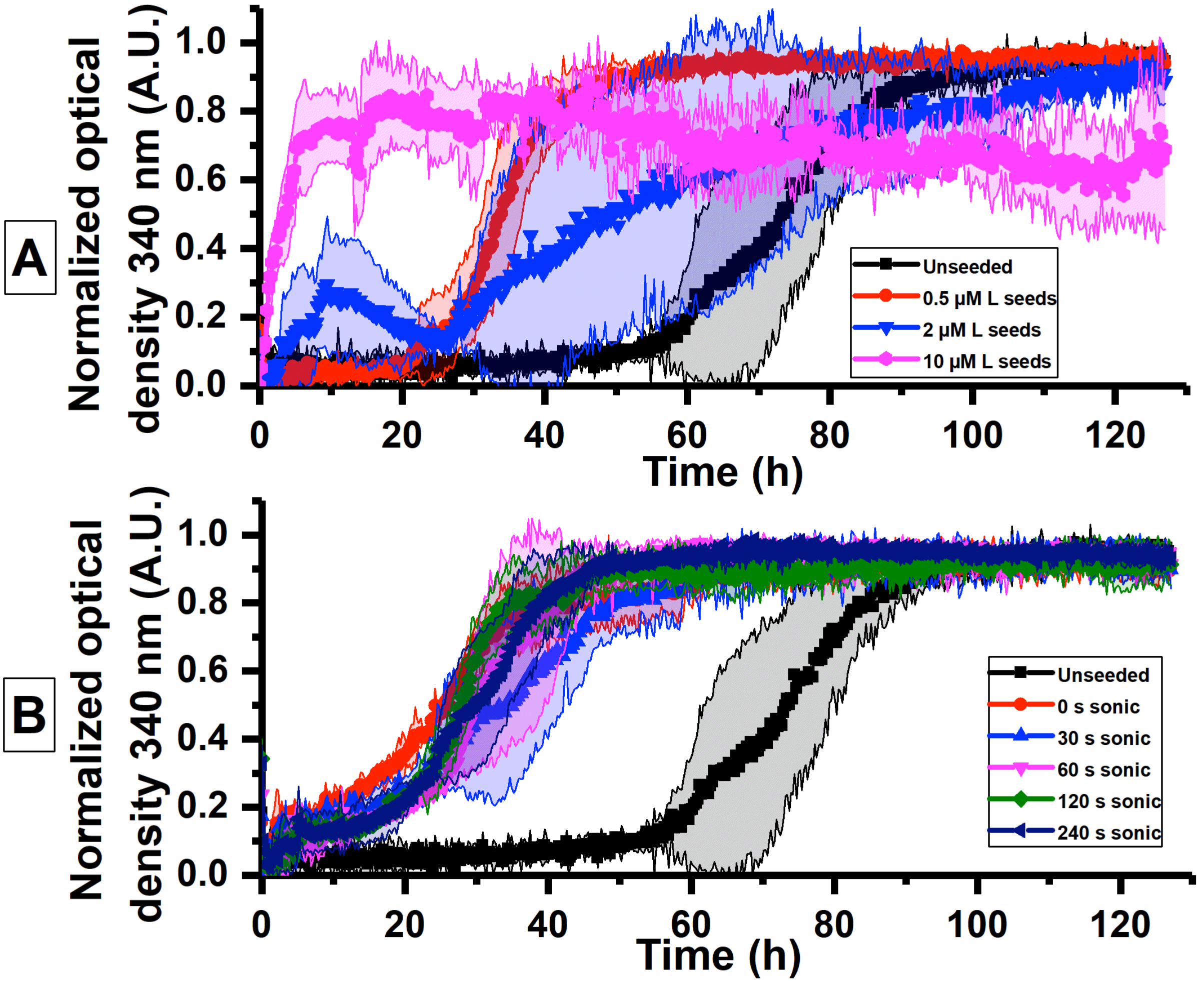

Figure S4: A surface-mediated nucleation mechanism is responsible for the heterologous seeding of aSyn aggregation by lysozyme fibrils. A) Aggregation kinetics of aSyn in the presence of different lysozyme seed ('L') concentrations (equivalent monomer) ; B) Aggregation kinetics of aSyn in the presence of 2 µM lysozyme seed concentration (equivalent monomer). After forming lysozyme fibrils, these were treated at different sonication times (0 s, 30 s, 60 s, 120 s and 240 s of sonication). Error bars denote standard deviations among triplicates.
